# Gene-Based Therapeutics for Acquired Retinal Disease: Opportunities and Progress

**DOI:** 10.3389/fgene.2021.795010

**Published:** 2021-12-07

**Authors:** Tien-En Tan, Beau James Fenner, Veluchamy Amutha Barathi, Sai Bo Bo Tun, Yeo Sia Wey, Andrew Shih Hsiang Tsai, Xinyi Su, Shu Yen Lee, Chui Ming Gemmy Cheung, Tien Yin Wong, Jodhbir Singh Mehta, Kelvin Yi Chong Teo

**Affiliations:** ^1^ Singapore National Eye Centre, Singapore, Singapore; ^2^ Singapore Eye Research Institute, Singapore, Singapore; ^3^ Duke-National University of Singapore Medical School, Singapore, Singapore; ^4^ Department of Ophthalmology, Yong Loo Lin School of Medicine, National University of Singapore, Singapore, Singapore; ^5^ Institute of Molecular and Cell Biology (IMCB), Agency for Science, Technology and Research (A*STAR), Singapore, Singapore; ^6^ Department of Ophthalmology, National University Hospital, Singapore, Singapore

**Keywords:** gene therapy, genome editing, ocular biofactory, neovascular age related macular degeneration (nAMD), geographic atrophy (GA), diabetic retinopathy (DR), diabetic macular edema (DME), retinal vascular disease

## Abstract

Acquired retinal diseases such as age-related macular degeneration and diabetic retinopathy rank among the leading causes of blindness and visual loss worldwide. Effective treatments for these conditions are available, but often have a high treatment burden, and poor compliance can lead to disappointing real-world outcomes. Development of new treatment strategies that provide more durable treatment effects could help to address some of these unmet needs. Gene-based therapeutics, pioneered for the treatment of monogenic inherited retinal disease, are being actively investigated as new treatments for acquired retinal disease. There are significant advantages to the application of gene-based therapeutics in acquired retinal disease, including the presence of established therapeutic targets and common pathophysiologic pathways between diseases, the lack of genotype-specificity required, and the larger potential treatment population per therapy. Different gene-based therapeutic strategies have been attempted, including gene augmentation therapy to induce *in vivo* expression of therapeutic molecules, and gene editing to knock down genes encoding specific mediators in disease pathways. We highlight the opportunities and unmet clinical needs in acquired retinal disease, review the progress made thus far with current therapeutic strategies and surgical delivery techniques, and discuss limitations and future directions in the field.

## Introduction

The success of gene augmentation therapy for monogenic inherited retinal disease (IRD) has been a significant milestone in establishing gene-based therapeutics as an important new treatment strategy for a wide range of inherited and acquired diseases in medicine. Voretigene neparvovec (Luxturna; Spark Therapeutics, Inc., Philadelphia, PA, USA) was the first Food and Drug Administration (FDA)-approved gene therapy for treatment of an inherited genetic disease (Russell et al., 2017). Voretigene neparvovec is administered via a single subretinal injection, and uses an adeno-associated viral (AAV) vector serotype 2 (AAV2) to restore a functional copy of the *RPE65* gene in patients with biallelic mutations in *RPE65* causing retinal disease. The successful demonstration of this therapeutic strategy has paved the way for numerous other clinical trials employing a similar gene augmentation approach to treat monogenic IRDs. Clinical trials are ongoing for many other IRDs, including choroideremia, retinitis pigmentosa (RP), achromatopsia, X-linked retinoschisis and Stargardt disease (Takahashi et al., 2018).

Gene-based therapy for monogenic IRDs is particularly exciting because until recently, these rare but devastating diseases were considered untreatable. However, there are some inherent limitations in the field. Although monogenic IRDs overall affect about 1 in 3,000 individuals, they are genotypically and phenotypically diverse, and are individually rare (Tan et al., 2021). Pathogenic variants in more than 300 genes have been implicated in monogenic IRDs, and gene-based therapies in this context are genotype-specific. Therefore, the feasibility and cost-effectiveness of developing and validating specific treatments for rarer mutations with small absolute numbers of affected individuals can be a significant challenge. It may be that rarer mutations or variants fall below the threshold for economic viability. In contrast, acquired (or multifactorial, complex, polygenic) retinal diseases such as age-related macular degeneration (AMD), and diabetic retinopathy (DR) represent a huge opportunity for the potential application of new therapeutic strategies, as the numbers affected by these diseases are much larger. It is estimated that AMD, the fourth leading cause of blindness worldwide, affects more than 190 million individuals around the world (Wong et al., 2014; Flaxman et al., 2017). Likewise, DR, a leading cause of blindness in the working age population, affects more than 100 million individuals worldwide (Yau et al., 2012; Flaxman et al., 2017; Wong et al., 2018). The prevalence rates of both these conditions are set to rise with aging global populations. Taken together, both neovascular AMD and DR can be considered retinal angiogenic diseases (RADs). This group of conditions also includes retinal vein occlusions and together, RADs make up the majority of treatable retinal disease today. Due to similarities in pathophysiological pathways, common therapeutic targets have been identified in these diseases, which provides potential for a genotype-agnostic or “one-size-fits-all” approach. One such key target is vascular endothelial growth factor (VEGF), and anti-VEGF therapy is the current standard of care for the treatment of RADs (Miller et al., 1994). Therefore, acquired retinal disease, especially RAD, represents a major opportunity to translate the discoveries and breakthroughs made in gene-based therapies for monogenic IRDs, to tackle these major causes of acquired visual loss worldwide.

In this review, we discuss the progress that has been made in applying gene-based therapeutics to acquired retinal disease thus far, highlight some of the key differences that separate it from the monogenic IRD field, and outline the major therapeutic and delivery strategies, limitations, and future directions.

## Inherited Versus Acquired Retinal Disease

In attempting to translate some of the advances in gene-based therapeutics from monogenic IRDs to multifactorial acquired retinal disease, it is useful to appreciate some of the differences between the two fields. Some of these key differences are summarized in Table 1. In general, gene-based therapies for monogenic IRDs are more specific, requiring targeting of specific genetic variants, pathways, and cell types/locations (Garoon and Stout, 2016; Takahashi et al., 2018; Cho et al., 2019; Xu et al., 2021). This limits the potential treatment population for each therapy developed, and is a driver of increased cost. In contrast, applying gene-based therapeutics towards acquired retinal disease is likely to be more “agnostic,” relying more on common therapeutic targets (such as VEGF), and being less specific for particular cell types or locations (Xu et al., 2021). Consequently, such therapies, when developed, may have larger potential target populations—although it remains to be seen if this will translate to lower cost per treatment in practice.

**TABLE 1:** Key differences in the application of gene-based therapeutics for inherited and acquired retinal diseases.

Monogenic inherited retinal disease	Multifactorial acquired retinal disease
Genotype-specific; Relies on accurate identification of the causative genetic variant in individual patients	Genotype-agnostic; Does not require identification of causative genetic variants in individual patients
Therapeutic targets and pathophysiologic pathways are genotype-specific	Therapeutic targets and pathophysiologic pathways may not be disease-specific; e.g., VEGF pathway is common to neovascular AMD, DR, RVOs
Treatment and delivery are targeted at specific cell types and locations, e.g., photoreceptors at the macula	In “ocular biofactory” approaches, treatment and delivery do not have to be targeted at specific cell types or locations
Smaller treatment population per therapy developed; Translates to higher cost per treatment; However, may qualify for “orphan drug” designation	Larger potential treatment population per therapy developed; May translate to lower cost per treatment; Most diseases will not qualify for “orphan drug” designation
Possibility of prophylactic therapy, e.g., *in utero* or pre-implantation, only if diagnosis is suspected or known at that stage	Possibility of prophylactic therapy at earlier stages of disease, e.g., early or intermediate AMD, non-proliferative DR
Lower bar for acceptance in terms of safety and efficacy; Standard of care is largely expectant management	Higher bar for acceptance; Safety and efficacy need to be compared against standard of care treatment, e.g., intravitreal anti-VEGF therapy or laser photocoagulation
Blood-retinal barrier more likely to be intact; Less potential for systemic immunogenicity	Blood-retinal barrier may be compromised; May have greater potential for systemic immunogenicity

VEGF, vascular endothelial growth factor; AMD, age-related macular degeneration; DR, diabetic retinopathy; RVO, retinal vein occlusion.

While the prospects for gene-based therapeutics in acquired retinal disease are certainly attractive, it is important to point out that such therapies will have to meet a high standard for regulatory approval and clinical acceptance. They will need to be rigorously evaluated against existing therapies that are currently standard of care, and are only likely to achieve widespread adoption if they can demonstrate significant advantages over current solutions. In contrast, for monogenic IRDs which are traditionally considered untreatable, the threshold for regulatory approval and clinical acceptance is naturally much lower, and treatments are also more likely to qualify for “orphan drug” designation, which is advantageous. In addition, gene-based therapeutics for both IRDs and acquired retinal diseases are bound by discrete therapeutic windows. They are only likely to be effective before the onset of irreversible retinal structural damage, such as retinal atrophy or fibrotic scarring. At the point of such late-stage disease, alternative emerging strategies such as regenerative or cell-based therapies, will probably need to be pursued.

## Unmet Needs and Opportunities in Acquired Retinal Disease

### Age-Related Macular Degeneration

AMD is the fourth most common cause of blindness worldwide, and the most common cause in developed countries (Wong et al., 2014; Flaxman et al., 2017). Current estimates indicate that more than 190 million individuals have AMD globally, with prevalence rates expected to increase dramatically as global populations age. It is estimated that by 2040, this figure will rise to 288 million individuals (Wong et al., 2014). Late stage AMD consists of both geographic atrophy (GA) and neovascular AMD, but currently there are only effective treatments for neovascular AMD. The mainstay of neovascular AMD treatment for the past 15 years has involved intravitreal injections targeting VEGF, which is a major angiogenic factor implicated in the disease pathophysiology (Kim and D’Amore, 2012). Anti-VEGF treatment can produce dramatic improvements in visual outcomes for patients with neovascular AMD (Brown et al., 2006; Rosenfeld et al., 2006). However, injections need to be given frequently (as often as monthly) in order to realize these visual gains, and this high treatment burden is often unsustainable. As a result, real-world treatment of AMD largely fails to achieve the visual gains seen in the pivotal clinical trials (Chong, 2016; Mehta et al., 2018; Fenner et al., 2020). Under-treatment in real-world settings is due to multiple factors, including cost, patient and physician fatigue, and non-adherence to therapy or follow-up (Lad et al., 2014; Okada et al., 2021).

### Diabetic Retinopathy

DR is also a major acquired retinal disease where gene-based therapeutics have enormous potential. Diabetes mellitus is estimated to affect more than 450 million individuals worldwide, and this figure is also expected to increase rapidly in the coming decades (Saeedi et al., 2019). Of these, about 30–40% will have DR, and 5–10% will have vision-threatening complications (VTDR), which include severe non-proliferative DR, proliferative DR, and DME (Yau et al., 2012; Ruta et al., 2013; Sabanayagam et al., 2016; Wong and Sabanayagam, 2019). Complications of proliferative DR can result in devastating irreversible severe visual loss, while DME is the most common cause of visual impairment in DR (Bandello et al., 2017). Crucially, visual loss from these complications of DR is preventable, and treatable. Neovascular AMD, DME and proliferative DR share similar VEGF-driven pathophysiologic pathways, and VEGF inhibitors are effective as treatments for these conditions (Antonetti et al., 2012; Bandello et al., 2013; Writing Committee for the Diabetic Retinopathy Clinical Research Network et al., 2015; Cohen and Gardner, 2016; Pearce et al., 2020).

VEGF inhibitor therapy for DME/DR has shortcomings similar to that for neovascular AMD. The treatment regimens dictate frequent regular injections, are costly, and may not have long-term efficacy (Lin et al., 2016; Hutton et al., 2017; Pearce et al., 2020). Like in neovascular AMD, anti-VEGF therapy is now the first-line treatment for center-involved-DME, but the treatment burden required to achieve substantial visual gains is high (Wells et al., 2016). Real-world evidence suggests that patients receiving anti-VEGF therapy for DME are also frequently under-treated, and have lower visual gains than those seen in the pivotal clinical trials (Blinder et al., 2017; Mitchell et al., 2020; Van Aken et al., 2020).

Gene-based therapeutics could help to address many of these real-world issues in current standard of care treatment with VEGF inhibitors for RADs (neovascular AMD and DME/DR) (Moore et al., 2017; Xu et al., 2021). Such a treatment modality that can provide effective and durable VEGF blockade would be ideal. Potentially, with gene-based therapies, good outcomes could be achieved with less frequent treatments for patients with neovascular AMD and DME/DR. Various gene-based treatment strategies have been attempted for these diseases, including gene augmentation to induce endogenous production of therapeutic factors or the formation of “ocular biofactories,” gene editing with clustered regularly interspaced short palindromic repeats (CRISPR), and RNA interference (RNAi), which are discussed in more detail below (Moore et al., 2017; Xu et al., 2021). Current work that is in the stage of clinical trials, and which is the closest to successful translation, focuses on the use of gene augmentation to allow for endogenous production of therapeutic proteins, with most trials targeting VEGF suppression (Campochiaro et al., 2006; Rakoczy et al., 2015; Constable et al., 2016; Campochiaro et al., 2017; Constable et al., 2017; Heier et al., 2017; Moore et al., 2017; Rakoczy et al., 2019; Xu et al., 2021). Clinical trials utilizing this “ocular biofactory” approach for treatment of neovascular AMD, non-neovascular AMD, and DME/DR are summarized in Table 2. At present, there are fewer such clinical trials for DR and DME than there are for AMD.

**TABLE 2:** Clinical trials for “ocular biofactory” gene therapies in age-related macular degeneration and diabetic retinopathy.

Therapeutic molecule	Vector	Delivery strategy	Phase	Sponsor	Drug name (if available)	Clinical trial identifier(s)	Publications (if available)
Neovascular AMD							
PEDF	Adenovirus	IVT	I	GenVec	—	NCT00109499	Campochiaro et al. (2006)
Endostatin, angiostatin	Lentivirus (EIAV)	SR	I	Oxford Biomedica	RetinoStat	NCT01301443	Campochiaro et al. (2017)
sFlt-1	AAV2	IVT	I	Sanofi Genzyme	—	NCT01024998	Heier et al. (2017)
sFlt-1	AAV2	SR	I/IIa	Adverum Biotechnologies, Inc. (formerly Avalanche Biotechnologies, Inc.)	—	NCT01494805	Rakoczy et al. (2015); Constable et al. (2016); Constable et al. (2017); Rakoczy et al. (2019)
Aflibercept	AAV2.7m8	IVT	I	Adverum Biotechnologies, Inc.	ADVM-022	NCT03748784	—
						NCT04645212	
Monoclonal anti-VEGF fab	AAV8	SR or SC	I/IIa II IIb/III	REGENXBIO Inc.	RGX-314	NCT03066258	—
						NCT03999801	
						NCT04514653	
						NCT04704921	
						NCT04832724	
CD59	AAV2	IVT	I	Hemera Biosciences (rights now acquired by Janssen Pharmaceuticals, Inc.)	HMR59	NCT03585556	—
Non-neovascular AMD (Geographic atrophy)							
CD59	AAV2	IVT	I	Hemera Biosciences (rights now acquired by Janssen Pharmaceuticals, Inc.)	HMR59	NCT03144999	—
Complement factor I	AAV2	SR	I/II II	Gyroscope Therapeutics Limited	GT005	NCT03846193	—
						NCT04437368	
						NCT04566445	
Diabetic retinopathy (without DME)							
Monoclonal anti-VEGF fab	AAV8	SC	II	REGENXBIO Inc.	RGX-314	NCT04567550	—
DME							
Aflibercept	AAV2.7m8	IVT	II	Adverum Biotechnologies, Inc.	ADVM-022	NCT04418427	—

AAV2, adeno-associated virus serotype 2; AAV2.7m8, adeno-associated virus serotype 2 with 7m8 capsid protein; AAV8, adeno-associated virus serotype 8; AMD, age-related macular degeneration; DME, diabetic macular edema; EIAV, equine infectious anemia virus; fab, antigen-binding fragment; IVT, intravitreal; PEDF, pigment epithelium-derived factor; SC, suprachoroidal; sFlt-1, soluble fms-like tyrosine kinase 1 or soluble vascular endothelial growth factor receptor 1; SR, subretinal; VEGF, vascular endothelial growth factor.

### Other Acquired Retinal Disease Targets

Gene-based therapeutics that can provide long-lasting and effective VEGF blockade would presumably also be effective for other RADs. For example, VEGF inhibitors are also frequently used for treatment of macular edema in retinal vein occlusions (RVOs), and retinopathy of prematurity (ROP). Retinal vein occlusions are the second most common retinal vascular disorder after DR, and affect about 28 million individuals worldwide (Song et al., 2019). ROP is a sight-threatening pediatric retinal vascular disorder that is amenable to VEGF inhibitor therapy. However, because anti-angiogenic treatment is only required within a relatively short therapeutic window for ROP (until retinal vascularization is achieved), and given that there are concerns about long-term VEGF suppression in developing infants, gene-based therapeutics for this purpose may not be ideal (Mintz-Hittner et al., 2011; Stahl et al., 2019).

Proliferative vitreoretinopathy (PVR) is the most frequent cause of failure following retinal detachment surgery, and is a challenging complication to manage, which often results in poor visual outcomes (Claes and Lafetá, 2014; Idrees et al., 2019). PVR has been proposed as a potential target for gene-based therapeutics since the 1990s (Chaum and Hatton, 2002). Various therapeutic approaches have been suggested, including suicide gene therapy via the Herpes Simplex Virus (HSV) thymidine kinase pathway, and antisense oligonucleotide (ASO) therapy targeted against molecular mediators of PVR (Chaum and Hatton, 2002). However, thus far, such efforts have failed to materialize, largely because an effective therapeutic target has yet to be identified. PVR is a complex process involving multiple pathways, including inflammation, epithelial-mesenchymal transition, and fibrosis (Claes and Lafetá, 2014; Idrees et al., 2019; Delgado-Tirado et al., 2020). Numerous molecular mediators and cytokines involved in PVR have been identified, and many treatments targeted at these mediators have been attempted, but to date, there is no effective pharmacologic agent for prevention or reversal of PVR (Claes and Lafetá, 2014; Idrees et al., 2019). This failure highlights that the development of gene-based therapeutics for diseases requires thorough understanding of the pathophysiologic pathways involved, and ideally makes use of already established therapeutic targets.

## Gene-Based Therapeutic Strategies

### Gene Augmentation for Endogenous Production of Therapeutic Factors—The “Ocular Biofactory”

Gene augmentation involves using a gene therapy vector to add a protein-producing gene, either to replace the function of a missing gene (more specifically gene *replacement* therapy, which is used in autosomal recessive or X-linked monogenic IRDs), or to modify cellular function for therapeutic benefit (more specifically gene *addition* therapy) (Xu et al., 2021). In acquired retinal disease, gene augmentation or addition can allow for long-lasting, endogenous production of a therapeutic protein, which in effect creates an “ocular biofactory” (Moore et al., 2017; Xu et al., 2021). Because there are already effective and safe therapies for neovascular AMD, DR, and DME based on inhibition of VEGF, this approach to endogenously express and produce these therapeutic molecules in the eye is most promising. There are many clinical trials using this approach for AMD (both neovascular and non-neovascular forms), DR, and DME (Table 2). These trials largely utilize AAV vectors (of various serotypes) to insert the desired gene, with a few older trials utilizing adenoviral or lentiviral vectors (Campochiaro et al., 2006; Campochiaro et al., 2017). Early phase I clinical trials focused on upregulation of endogenous anti-angiogenic factors such as pigment epithelium-derived factor (PEDF), endostatin and angiostatin (Campochiaro et al., 2006; Campochiaro et al., 2017). Subsequently, with the success of VEGF inhibitor therapy, many newer trials have aimed to mimic this effect, either by expressing synthetic recombinant anti-VEGF proteins like aflibercept, or endogenous VEGF inhibitors such as soluble fms-like tyrosine kinase 1 (SFlt-1) (Rakoczy et al., 2015; Constable et al., 2016; Constable et al., 2017; Heier et al., 2017; Rakoczy et al., 2019). A few of these approaches are discussed in more detail below. In addition, some clinical trials are investigating expression of factors inhibiting the complement pathway, such as CD59 and complement factor I (CFI), mostly for non-neovascular AMD with GA.

SFlt-1 is a naturally-occurring endogenous VEGF inhibitor. Gene augmentation for endogenous therapeutic expression of SFlt-1 has been investigated by both intravitreal and subretinal delivery approaches (Rakoczy et al., 2015; Constable et al., 2016; Constable et al., 2017; Heier et al., 2017; Rakoczy et al., 2019). Intravitreal injection with an AAV2 vector was evaluated in a phase I clinical trial (Heier et al., 2017). The treatment was safe and well-tolerated, but expression levels of sFlt-1 and treatment response were variable. The authors suggested that baseline anti-AAV2 serum antibodies may have influenced efficacy (Heier et al., 2017). Subretinal delivery with an AAV2 vector has also been studied in phase I/IIa clinical trials, which demonstrated that the treatment was safe, and well-tolerated (Constable et al., 2016; Rakoczy et al., 2019). Efficacy data did not show a significant difference in visual acuity or retinal thickness, although the trial was not designed to evaluate this as a primary outcome measure (Constable et al., 2016; Rakoczy et al., 2019).

RGX-314 (REGENXBIO Inc., Rockville, MD, USA), which uses the endogenous therapeutic approach, is currently being evaluated for neovascular AMD and DR without DME. RGX-314 uses an AAV8 vector to induce production of a monoclonal anti-VEGF antigen-binding fragment (fab). This is delivered either by a subretinal or suprachoroidal approach. Phase I/IIa clinical trials have so far demonstrated that the treatment is well-tolerated, and safe (unpublished data) (Campochiaro, 2021; Ho, 2021). Unpublished data from phase I/IIa trials seems to suggest that RGX-314 may allow for stable visual acuity, reduced retinal thickness, and reduced anti-VEGF treatment burden (Campochiaro, 2021; Ho, 2021). Phase II (AAVIATE) and IIb/III (ATMOSPHERE) clinical trials are currently underway for neovascular AMD, and a phase II trial (ALTITUDE) is being conducted for DR without DME.

ADVM-022 (Adverum Biotechnologies, Inc., Redwood City, CA, USA) utilizes a similar approach for neovascular AMD and DME. ADVM-022 uses an AAV2.7m8 vector delivered via an intravitreal injection, to induce endogenous production of aflibercept in the eye. Two-year outcomes from the phase I OPTIC trial suggest that the treatment is well tolerated, with a low incidence of intraocular inflammation requiring topical steroids (unpublished data) (Khanani, 2021). Intraocular production of aflibercept seems to be maintained up to 2 years, with stable visual acuity, reduced retinal thickness, and reduced anti-VEGF treatment burden (unpublished data) (Khanani, 2021). Phase III clinical trials for neovascular AMD are planned, and a phase II clinical trial (INFINITY) for DME is ongoing.

### Gene Editing With Clustered Regularly Interspaced Short Palindromic Repeats Technology

CRISPR technology, combined with various bacterial nucleases, is able to achieve highly precise and targeted editing of genes at specific loci *in vivo*. This has been proposed as a potential treatment modality for neovascular AMD, DME and other acquired RADs by employing gene editing to knock down various mediators in the angiogenic pathways (Lin et al., 2020).

Thus far, such gene editing strategies for acquired retinal disease have only been explored in the preclinical stage, largely in murine models of choroidal neovascularization (CNV). Reported approaches have used CRISPR together with Cas9 nucleases from *Streptococcus pyogenes* (SpCas9) or *Campylobacter jejuni* (CjCas9), as well as RNA-guided endonuclease from Lachnospiraceae bacterium (LbCpf1) (Holmgaard et al., 2017; Huang et al., 2017; Kim E. et al., 2017; Kim K. et al., 2017; Koo et al., 2018; Lin et al., 2020; Ling et al., 2021). Most studies have employed viral vectors such as AAV and lentivirus for delivery of nucleases. However, some groups have tried avoiding viral vectors, instead directly administering pre-assembled Cas9 ribonucleoproteins (RNPs), which may reduce problems with immune and antibody responses (Kim K. et al., 2017). Vectors are delivered either by subretinal or intravitreal injections, and the genes targeted include those responsible for production of VEGF receptor 2 (VEGFR2), VEGF-A and hypoxia inducing factor-1α (HIF-1α) (Holmgaard et al., 2017; Huang et al., 2017; Kim E. et al., 2017; Kim K. et al., 2017; Koo et al., 2018; Lin et al., 2020; Ling et al., 2021). In theory, these gene editing techniques should also provide for durable long-term suppression of angiogenesis. However, the potential for “off-target effects” of gene editing causing mutations at other sites is a risk that needs to be considered and minimized (Zhang et al., 2015). Evaluation of these techniques in human clinical trials for neovascular AMD and other acquired RADs is anticipated.

### Other Strategies

RNAi is a proposed therapeutic modality that uses specifically designed, short sequences of RNA such as small interfering RNA (siRNA), short hairpin RNA (shRNA) or micro RNA (miRNA) to bind to and degrade messenger RNA (mRNA), to silence expression of targeted genes (Weng et al., 2019). RNAi has been investigated for treatment of neovascular AMD since the 2000s, with preclinical and clinical trials evaluating intravitreal injections of siRNA designed to block VEGF activity (Weng et al., 2019). Bevasiranib and Sirna-027 (AGN211745) are two prominent examples that were evaluated in clinical trials. Bevasiranib was designed to silence VEGF production, and was evaluated up to phase III clinical trials. Unfortunately, the treatment effect was disappointing, and the trial was terminated (Garba and Mousa, 2010; Weng et al., 2019). Sirna-027 (AGN211745) was designed to target VEGF receptor 1 (VEGFR1), and was evaluated in a phase II clinical trial, that was also eventually terminated due to inadequate therapeutic effect (Weng et al., 2019). Thereafter, the approach using RNAi for treatment of neovascular AMD was also largely abandoned as it was shown that the effect of CNV regression seen in preclinical models was not from targeted inhibition of the VEGF pathway as previously thought (Kleinman et al., 2008). Furthermore, such RNAi approaches would not have provided durable treatment effect, and would also have required frequent or repeated intravitreal injections. In recent years, however, there has been some renewed interest in the technology, with some groups investigating the use of gene augmentation therapy with viral vectors to induce endogenous production of shRNA and miRNA against VEGF via an endogenous therapeutic approach (Askou et al., 2012; Askou et al., 2015; Askou et al., 2019). This approach should, in theory, provide durable treatment effect, but thus far has only been evaluated in preclinical studies.

Optogenetics is an approach that attempts to generate new photosensitive cells in the retina in patients with advanced atrophy and photoreceptor loss (Pan et al., 2015). Viral vectors are used to introduce genes encoding photosensitive proteins, to convert retinal cells (other than photoreceptors) into photosensitive cells. This approach is genotype- and disease-agnostic, and has the potential to provide some functional visual restoration in patients with advanced or end-stage retinal disease. GS030 (GenSight Biologics S.A., Paris, France) is an optogenetic treatment that uses an intravitreal AAV2.7m8 vector to induce production of photosensitive ChrimsonR protein in retinal ganglion cells (RGCs). Recently, it was reported that GS030 treatment, together with specially engineered light-stimulating goggles, was able to provide partial functional recovery in a patient with advanced RP (Sahel et al., 2021). GS030 is currently being evaluated in a phase I/IIa clinical trial for RP. Similarly, RST-001 (Allergan Inc., Irvine, CA, USA) uses an intravitreal AAV2 vector to induce production of photosensitive channelrhodopsin-2 protein in RGCs, and is being evaluated in a phase I/IIa clinical trial for advanced RP (Moore et al., 2017). If these optogenetic therapies prove successful in RP, they could potentially be applied to advanced AMD and other acquired retinal diseases as well.

Another interesting gene-based regenerative medicine approach that has potential in late-stage acquired retinal disease is epigenetic reprogramming with the Oct4, Sox2 and Klf4 (OSK) system (Lu et al., 2020). This approach focuses on inducing expression of the OSK genes in target cells with viral vectors, to reverse age-related DNA methylation, and “reprogram” somatic cells to allow for *in vivo* regeneration. Recently, it was demonstrated that OSK genes delivered by intravitreal injection of AAV vectors in a mouse model of glaucoma were able to induce regeneration of RGC axons and reverse vision loss (Lu et al., 2020). It remains to be seen if this technique can be useful for outer retinal regeneration as well, and if these results can be reproduced in other models.

## Surgical Delivery Strategies

### Intravitreal Injections

Intravitreal injections are attractive as a delivery strategy for gene-based therapeutics for a number of reasons. They are quick, safe, office-based procedures that can be performed under topical anesthesia by general ophthalmologists. They do not require expensive specialized equipment, and are regularly performed already for treatment of many acquired retinal diseases. They can also be easily repeated, if necessary. In the context of gene-based therapeutics, delivery into the vitreous cavity also has the potential for transduction of the whole retina and the ciliary body, which may be ideal if the aim is to induce maximal levels of endogenous expression.

**FIGURE 1:**
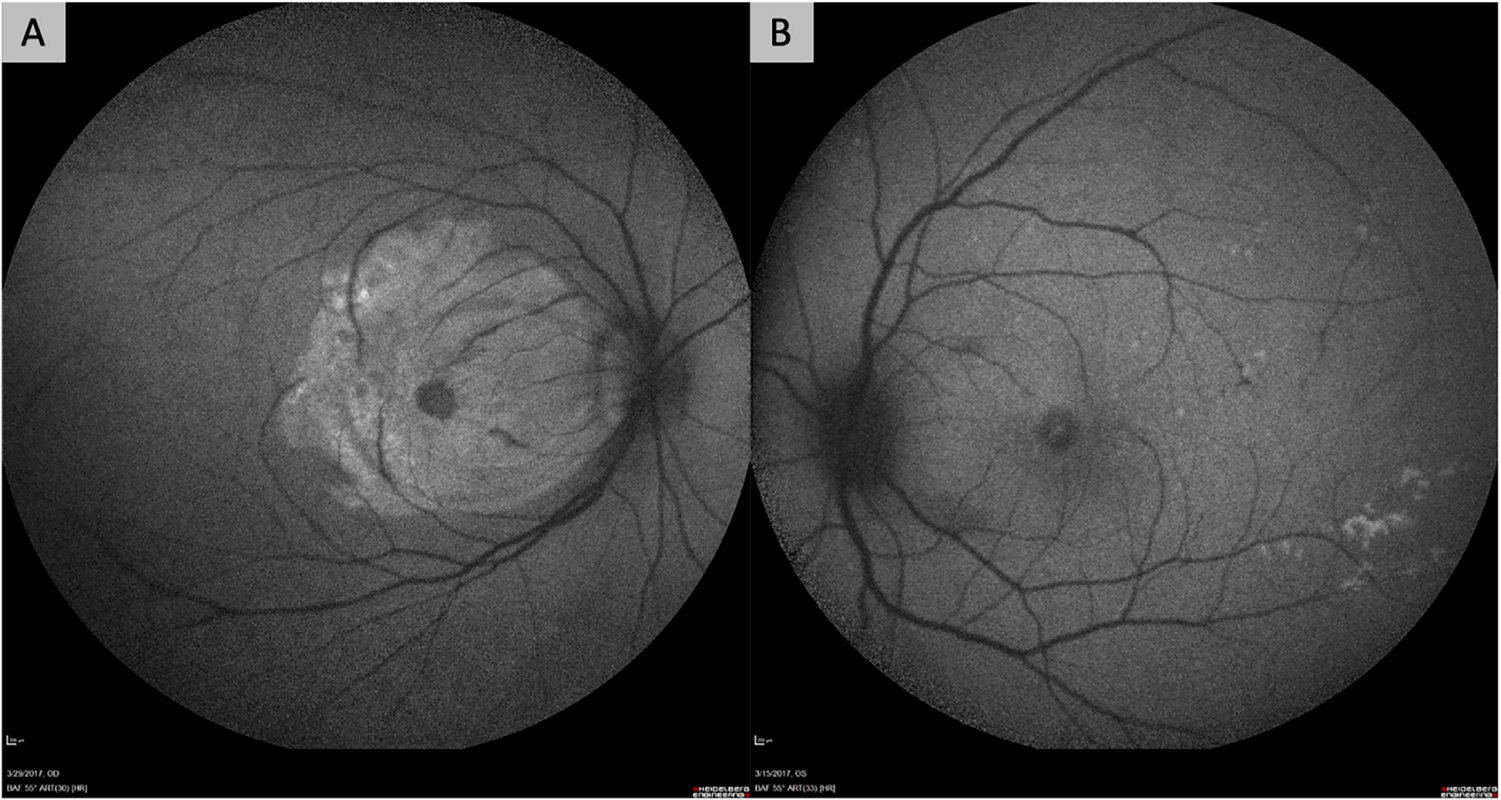
Fundus autofluorescence images of non-human primate eyes demonstrating the improved efficacy of retinal gene transfection after surgical removal of the internal limiting membrane (ILM). Both eyes in the figure had intravitreal delivery of AAV2 vector carrying the gene for green fluorescent protein (GFP) after a pars plana vitrectomy. One eye **(A)** had surgical peeling of the ILM over the macula prior to exposure to the AAV2 vector, while the other eye **(B)** did not. The eye that underwent ILM removal **(A)** shows significant hyper-autofluorescence over the macular region from GFP expression, while the other does not **(B)**.

However, the delivery of gene-based therapies via intravitreal injection has some important drawbacks. First, transduction of the outer retina, photoreceptors, and RPE may be limited by the ability of viral vectors to cross the vitreous, internal limiting membrane (ILM) and inner retinal layers. These structures in the eye are hypothesized to be physical and biological barriers to transfection of the outer retina and RPE. In particular, frequently-used AAV vectors, such as AAV2, have limited ability to cross the ILM. Suggested solutions to this have included the use of directed evolution techniques to identify serotypes with greater ability to cross the ILM, such as AAV2.7m8 (which is utilized by ADVM-022), or surgical removal of the ILM prior to intravitreal injection of vectors (Dalkara et al., 2013; Khabou et al., 2016; Takahashi et al., 2017; Teo et al., 2018). Our group found that surgical removal of the ILM prior to exposing the retina to vector resulted in more effective transfection, even with the AAV2 serotype (Figure 1) (Teo et al., 2018).

Second, the presence and development of neutralizing antibodies (NAbs) to viral vectors in the vitreous cavity is a major hurdle. The presence of pre-existing NAbs against viral vectors results in vitreous inflammation, and poorer transduction efficacy (Heier et al., 2017; Xu et al., 2021). Such pre-existing NAbs can occur due to natural infection with wild-type AAV, and it has been shown that the prevalence of such NAbs increases with age (Calcedo et al., 2011). Furthermore, intravitreal injection of AAV vectors can induce development of NAbs, which can limit the efficacy of re-treatments, and also show cross-reactivity across different serotypes (Kotterman et al., 2015). Multiple studies in both non-human primates and humans have demonstrated that delivery of viral vectors into the vitreous cavity results in activation of the adaptive immune system, and triggers a significant humoral response (Kotterman et al., 2015; Reichel et al., 2018).

### Subretinal Injections

At present, subretinal injections are the most well-established delivery route for gene-based therapeutics (Xu et al., 2021). Voretigene neparvovec is in clinical use, and is delivered by a subretinal injection after pars plana vitrectomy (Russell et al., 2017). Numerous other investigational gene therapies, including RGX-314, RetinoStat (Oxford Biomedica, Oxford, UK), and treatments using SFlt-1 also utilize a similar subretinal approach. The subretinal approach is advantageous as it has greater immune-privilege than the intravitreal approach. Viral vectors injected into the subretinal space cause less inflammation, do not induce the production of NAbs, and may be more amenable to re-treatments for these reasons (Rakoczy et al., 2015; Reichel et al., 2018; Xu et al., 2021). If the outer retina, photoreceptors, or RPE are the desired sites of transduction, viral vectors in the subretinal space are also likely to have greater transduction efficacy because of direct contact and close proximity. Furthermore, subretinal injections can be targeted at specific retinal locations, such as the macula (Xu et al., 2021).

Disadvantages of the subretinal approach include the need for a pars plana vitrectomy, which limits the procedure to vitreoretinal surgeons, is more costly for patients, and exposes them to greater risk of surgical complications, such as iatrogenic retinal breaks, rhegmatogenous retinal detachments, and cataract formation (Xu et al., 2021). Successful subretinal injection also has a steeper surgical learning curve. Control of bleb propagation can be challenging, and reflux of vector into the vitreous cavity can result in more inflammation, and reduce therapeutic efficacy. Treatment of the foveal region involves iatrogenic detachment of the fovea, which may have deleterious effects on foveal photoreceptors, and can also induce iatrogenic macular holes (Russell et al., 2017; Davis et al., 2019; Xu et al., 2021). Subretinal injections also only treat localized areas, and cannot achieve panretinal transduction.

As subretinal injections become more commonplace, surgical techniques have improved. Innovations that have improved the safety and reproducibility of the technique include the use of intraoperative optical coherence tomography (OCT) to allow accurate localization of the subretinal space, creation of pre-blebs with balanced salt solution to prevent reflux and wastage of the drug, and the use of foot pedal-controlled pneumatic injectors (viscous fluid control), which eliminates variability in injection pressure that may occur when an assistant handles the syringe (Davis et al., 2019; Xu et al., 2021).

### The Suprachoroidal Space

The suprachoroidal space between the choroid and sclera is a potential space that is being evaluated for therapeutic delivery to the retina and RPE (Kansara et al., 2020; Xu et al., 2021). Gene-based therapeutics can be delivered to the suprachoroidal space by free-hand injection through the sclera, guarded microneedle injection, or tunneled microcatheters (Kansara et al., 2020; Xu et al., 2021). Guarded microneedles are commercially available (Clearside Biomedical, Alpharetta, GA, USA), and have been used in clinical trials for delivery of triamcinolone into the suprachoroidal space (Campochiaro et al., 2018; Yeh et al., 2020). RGX-314 is being delivered by suprachoroidal microneedle injection in phase II clinical trials for both neovascular AMD and DR.

Microcatheters are surgically introduced into the suprachoroidal space via scleral cutdowns, and can be advanced to the posterior pole or other desired delivery sites in the posterior segment with surgical visualization via indirect ophthalmoscopy (Peden et al., 2011; Heier et al., 2020). A commercial suprachoroidal microcatheter has been developed that also allows therapeutic delivery into the subretinal space using an extendable microneedle coming from the suprachoroidal space (Orbit Subretinal Delivery System; Gyroscope Therapeutics Limited, London, UK). This approach allows for subretinal injection without a pars plana vitrectomy or retinotomy. This device is being evaluated for surgical delivery of GT005 (Gyroscope Therapeutics Limited), which is an AAV2 gene therapy for complement factor I in AMD with GA.

The suprachoroidal space is potentially advantageous because it allows for treatment of a larger area of retina than subretinal injections, and can in theory achieve panretinal transduction. It also bypasses the ILM barrier to the retina, does not require a pars plana vitrectomy, and can be done as an office-based procedure (Kansara et al., 2020; Xu et al., 2021).

## Limitations and Future Directions

Clearly, gene-based therapeutics have enormous potential for the treatment of acquired retinal disease. However, there are some important limitations that need to be acknowledged, and addressed in future research.

First, even if therapeutic strategies such as gene augmentation to produce an “ocular biofactory,” or gene editing with CRISPR, are genotype-agnostic for acquired retinal disease, they still rely on identification of appropriate therapeutic targets in disease pathophysiology. Such treatment strategies targeting angiogenesis in neovascular AMD and DR are only possible because the pathophysiologic pathways responsible have been well-studied and elucidated. Therefore, more basic research into the molecular mediators and pathways of acquired retinal disease is still crucial, so that new therapeutic targets can be identified for new gene-based therapeutics. The development of more robust and representative animal models of acquired retinal disease will also allow for more effective translation of these therapies.

Second, “ocular biofactory” or gene editing approaches are still bound by discrete therapeutic windows, and need to be instituted before the development of significant retinal scarring or atrophy. Future treatment of end-stage retinal disease is likely to require alternative disease-agnostic approaches, such as optogenetic therapy, cell-based regenerative therapies, retinal protheses, or some combination of these approaches to restore visual function.

Third, the presence of NAbs against viral vectors and the induction of humoral immune responses are an important barrier to overcome, particularly with treatments administered by intravitreal injection. The potential for cross-reactivity of NAbs across viral serotypes is particularly problematic. Patients currently being enrolled in gene-based clinical trials do need to be made aware of this risk, and that the induction of NAbs may reduce the effectiveness of other potential future treatments that become available in future.

Fourth, one of the key advantages of gene-based therapeutics is the potential for durable treatment effect. However, prolonged treatment effect and irreversibility can be a double-edged sword. For example, it has been suggested by animal studies and some clinical trials that frequent treatment with VEGF inhibitor therapy may promote the development of retinal atrophy or GA (Saint-Geniez et al., 2009; Grunwald et al., 2014; Gemenetzi et al., 2017; Sadda et al., 2020; Chong Teo et al., 2021). Though the exact relationship between VEGF inhibition and development of retinal atrophy has yet to be established, it may be that chronic prolonged inhibition of VEGF after achieving disease quiescence is undesirable. Development of effective gene regulation technology may help to address this limitation. Reversible regulation of transgene expression has been described by molecules that can be administered topically or orally (Stieger et al., 2006; Le Guiner et al., 2014; Sochor et al., 2015; O’Callaghan et al., 2017). Reliable gene regulation technology in future could allow for transgenes to be effectively turned “on” when treatment is required, and “off” when disease stability is achieved.

## Conclusion

Acquired retinal diseases such as neovascular AMD, DR, and DME represent an enormous opportunity for new gene-based therapeutics. Translating our learnings from gene augmentation therapy and gene editing in monogenic IRDs could help to address many of the unmet clinical needs in the treatment of acquired retinal disease. Currently, the most promising approaches focus on gene augmentation therapy to create ocular biofactories for production of therapeutic molecules, and evaluation of this strategy in clinical trials is already underway. Further progress in the field will be driven by continued research into the mediators and mechanisms underlying disease pathophysiology to identify new therapeutic targets, and in optimization of treatment delivery strategies.
